# A Study on the Detection of Cattle in UAV Images Using Deep Learning

**DOI:** 10.3390/s19245436

**Published:** 2019-12-10

**Authors:** Jayme Garcia Arnal Barbedo, Luciano Vieira Koenigkan, Thiago Teixeira Santos, Patrícia Menezes Santos

**Affiliations:** 1 Embrapa Agricultural Informatics, Campinas-SP 13083-886, Brazil; luciano.vieira@embrapa.br (L.V.K.); thiago.santos@embrapa.br (T.T.S.); 2 Embrapa Southeast Livestock, São Carlos 13560-970, São Paulo, Brazil; patricia.santos@embrapa.br

**Keywords:** unmanned aerial vehicles, drones, canchim breed, nelore breed, convolutional neural networks

## Abstract

Unmanned aerial vehicles (UAVs) are being increasingly viewed as valuable tools to aid the management of farms. This kind of technology can be particularly useful in the context of extensive cattle farming, as production areas tend to be expansive and animals tend to be more loosely monitored. With the advent of deep learning, and convolutional neural networks (CNNs) in particular, extracting relevant information from aerial images has become more effective. Despite the technological advancements in drone, imaging and machine learning technologies, the application of UAVs for cattle monitoring is far from being thoroughly studied, with many research gaps still remaining. In this context, the objectives of this study were threefold: (1) to determine the highest possible accuracy that could be achieved in the detection of animals of the Canchim breed, which is visually similar to the Nelore breed (*Bos taurus indicus*); (2) to determine the ideal ground sample distance (GSD) for animal detection; (3) to determine the most accurate CNN architecture for this specific problem. The experiments involved 1853 images containing 8629 samples of animals, and 15 different CNN architectures were tested. A total of 900 models were trained (15 CNN architectures × 3 spacial resolutions × 2 datasets × 10-fold cross validation), allowing for a deep analysis of the several aspects that impact the detection of cattle using aerial images captured using UAVs. Results revealed that many CNN architectures are robust enough to reliably detect animals in aerial images even under far from ideal conditions, indicating the viability of using UAVs for cattle monitoring.

## 1. Introduction

Unmanned aerial vehicles (UAVs), also known as unmanned aerial systems (UAS) and drones, are becoming commonplace in agriculture. New applications are being constantly proposed, both in the crop and livestock production chains [1,2]. In the specific case of cattle monitoring, there are a few applications in which UAVs can have immediate impact, including the estimation of the number of animals, monitoring of anomalous events (diseased animals, calf birth, etc.) and measurement of body traits. All of those applications have one step in common: before the extraction of more sophisticated information, the animals need to be detected in the images. The definition of the term “detection” will often depend on the approach being used. In this work, convolutional neural networks (CNNs) architectures are used as classifiers, which means that the term “detection” refers to the recognition that a given image block contains at least part of an animal. It is worth noting that there are other ways to detect animals, such as using algorithms that estimate bounding boxes around the objects [3,4], and algorithms that delineate the animal bodies through semantic segmentation [5,6]. The main shortcoming of the former is that each architecture has some conditions under which they tend to perform poorly (e.g., you only look once (YOLO) [7] struggles with groups of small objects and to generalize aspect ratios), while the latter requires careful image annotation which can be very time-consuming. Because of those issues, they were not considered in this study.

Most of the work on the use of UAVs for animal monitoring has been dedicated to wild animals [8], including deer [9,10,11], elk [9], hippopotamus [12], rhinoceros [13] and elephants [14]. Although in the last few years the number of studies dedicated to cattle has risen, they are still relatively rare. A few academic investigations on this subject have been dedicated to animal detection and counting [15,16,17,18,19], cattle round-up [20], feeding behavior [21], animal identification [22] and health monitoring [23]. Most of those studies (especially the most recent ones) employ deep learning for animal detection.

All methods for cattle detection using deep learning found in the literature use one of two methods. The first approach uses CNNs to generate a probability heat map which hopefully shows where animals are located [15,17,18]. The second approach uses techniques that generate a bounding box around the objects of interest [19,22]. Both of these studies use the YOLO v2 architecture [7], which was designed with speed in mind. Although the results reported in all those studies are very encouraging, the experimental designs have some limitations that make it difficult to evaluate the real significance of the reported results. In particular, it seems that the aerial images were always captured under ideal conditions, but these conditions are not described and factors such as flight altitude and ground sample distance (GSD) associated, illumination, time of day, presence of shadows, etc., are not investigated. As a result, accuracies under practical operation conditions may be significantly lower, and since no recommendations or protocols are suggested to guide mission planning, avoiding problematic conditions may be challenging. It is also worth noting that, in the case of the first approach, the CNN architectures used in those were designed specifically for the problem at hand, but comparisons with more well-established architectures are limited or non-existent.

Most of the experimental limitations mentioned above are probably due to the fact that these are the first investigations dedicated to cattle detection, so they are more concerned with proving the concept than with the details that would make practical operation feasible. Nevertheless, expanding the understanding of the subject is essential for the future adoption of this type of technology, which is the main objective of this work. It is important to highlight that estimating the number of animals while being heavily dependent on effective methods for animal detection, require that other factors be taken into account (animal movement, image matching, etc.). This study was dedicated exclusively to the detection part of the counting problem, so those additional factors were not addressed.

This article brings four novel contributions:

1. All experiments were done with animals of the Nelore (*Bos indicus*) and Canchim breed. The Canchim breed is a cross between Charolais (*Bos taurus*) and Nelore breeds, with the latter lending most of its visual traits. To the authors’ knowledge, there are no studies in the literature using aerial images of either Nelore or Canchim breeds. Because visual differences between both breeds were slight in most cases, breed identification using the CNNs was not attempted.
2. Some experiments were designed specifically to determine the ideal GSD when CNNs are used, taking into consideration both accuracy and area covered.
3. Fifteen of the most successful CNN architectures were compared, using a 10-fold cross-validation procedure to avoid any spurious or unrealistic results.
4. The image dataset used in the experiments include images captured under a wide variety of conditions. To guarantee as much data variability as possible, images were captured under different weather conditions (sunny and overcast), at different times of the day and of the year, and with different pasture conditions. Each one of those factors is carefully analyzed and discussed, thus qualifying the results observed.

## 2. Materials and Methods

### 2.1. Image Dataset

**Figure 1:**
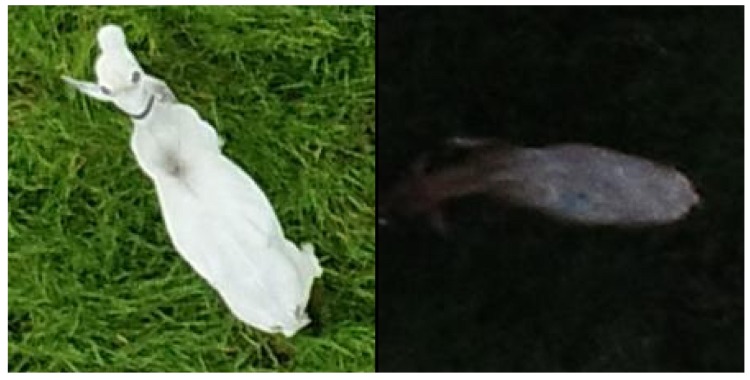
Examples of well-lit (**left**) and too dark conditions (**right**).

**Figure 2:**
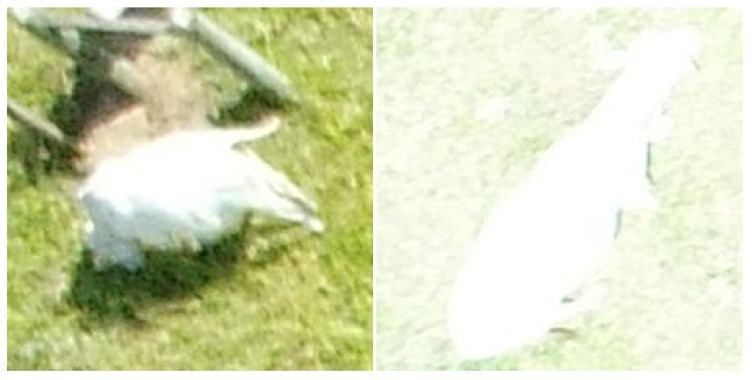
Examples of blurry (**left**) and too bright conditions (**right**).

**Figure 3:**
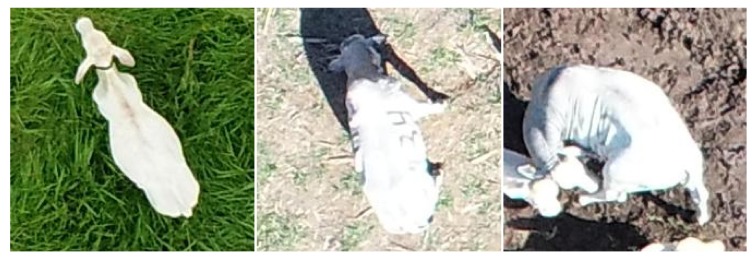
Examples of lush pasture (**left**), dry pasture (**center**) and exposed soil (**right**).

**Figure 4:**
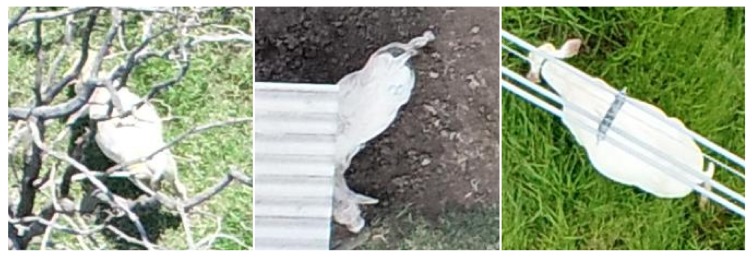
Examples of animal oclusions: tree branches (**left**), shed roof (**middle**), electrical wires (**right**).

The UAV used in the experiments was a DJI Phantom 4 Pro, equipped with an 20-MPixel camera. The 4:3 aspect ratio was adopted in the experiments, resulting in images with 4864 x 3648 pixels. Missions were carried out at the Canchim farm, São Carlos, Brazil (2158${}^{\prime }$28${}^{\prime \prime }$ S, 4750${}^{\prime }$59${}^{\prime \prime }$ W) at 11 dates over the year of 2018. Camera settings were all kept on automatic, except exposition, which used the presets “sunny” and “overcast” depending on weather conditions. Images were captured from an altitude of 30 m with respect to the take-off position. This altitude provided a fine GSD (approximately 1 cm/pixel) without disturbing the animals, which showed no reaction when the aircraft flew over them. Altitude and GSD had variations of up to 20% due to the ruggedness of the terrain. Frontal and side image overlap images were both set to 70%. Each animal is represented by 13,000 pixels in average—this number varies considerably with the size and position of the animals, as well as with the actual altitude at the moment of capture. The images cover a wide range of capture conditions, and animals from both Canchim and Nelore breeds were present during all flights. Illumination varied from well-lit to very dark conditions (Figure 1), which is due not only to the weather but also to condition variations in the same flight. The contrast between animals and background also varied significantly—low contrast situations were caused by both motion blur and excessive brightness (Figure 2). Because images were captured at different times of the year, soil conditions also varied (Figure 3). Different degrees of animal occlusions are also present (Figure 4). A total of 19,097 images were captured, from which 1853 images containing at least part of an animal were selected for the experiments. The maximum number of animals in a single image was 15.

**Figure 5:**
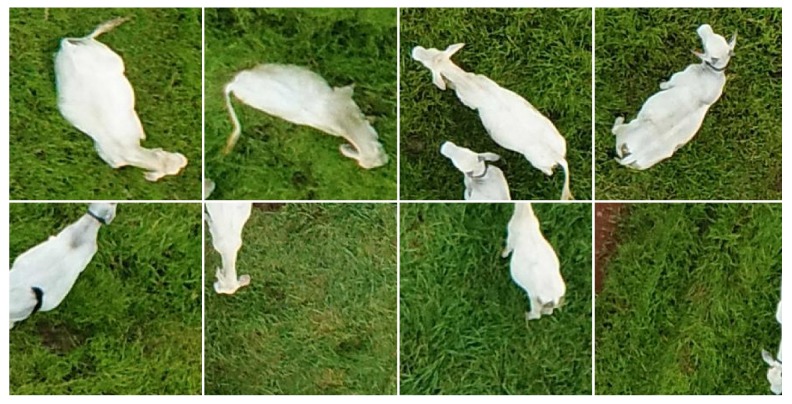
Examples of image blocks containing carefully framed animals (**top**) and image blocks generated using a regular grid (**bottom**).

Two datasets were generated from the original images. In the first one, 224 × 224 pixel squares were associated to all animals in all images, carefully encompassing each individual. Animals at the edges of the images were only considered if at least 50% of their bodies were visible. While the targeted animal was always centered, there were cases in which image blocks included parts of other animals due to close proximity. A total of 8629 squares containing animals were selected; the same number of squares was randomly selected to represent the background. This dataset was generated with the objective of determining the best possible accuracy that can be achieved when all objects of interest are perfectly framed by the image blocks to be classified. In the second dataset, images were subdivided into image blocks using a regular grid with 224-pixel spacing both horizontally and vertically. This value was chosen because 224 × 224 pixels is the default input size for many of the CNNs tested in this work and, additionally, blocks of this size encompass almost perfectly most of the animals present in the images. Blocks were then labeled as “cattle” and “non-cattle”; to be labeled as “cattle”, a block should contain at least a few pixels that could be undoubtedly associated with an animal without having any other block (or the entire image) as reference. This criterion is subjective and susceptible to inter- and intra-rater inconsistencies [24], but this was the most viable approach given the amount and characteristics of the images. A total of 14,489 image blocks were labeled as “cattle”; the number of “non-cattle” blocks was much higher but, in order to avoid problems associated with class imbalance, only 14,489 randomly selected “non-cattle” blocks were used in the experiments. Both image datasets were manually annotated by the same person. Some examples of “cattle” image blocks present in each dataset are shown in Figure 5.

### 2.2. Experimental Setup

**Figure 6:**
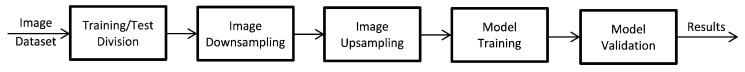
Workflow used to train all models considered in the experiments.

Figure 6 shows the basic workflow used to train each of the models tested in this study.

First, the original dataset containing all labeled image blocks was divided into a training (80% of the samples) and a test dataset (20%). A validation set was not used because the model hyperparameters were defined as having previous experiments as reference, as described later in this section. As a result, the training and test datasets always contained, respectively, 13,806 and 3452 samples when the first dataset was adopted (careful animal framing), and 23,182 and 5796 samples when the second dataset was used (regular grid). In order to avoid biased results due to skewed data distributions caused by the random dataset division, a 10-fold cross-validation was adopted. In other words, 10 models were trained for each pair of input dimension and CNN considered.

As mentioned before, all image blocks generated during the labeling process had 224 × 224 pixels. In order to simulate coarser GSDs, the original image blocks were downsampled to 112 × 112 pixels and 56 × 56 pixels, simulating GSDs of 2 and 4 cm/pixel or, equivalently, simulating flight altitudes of 60 and 120 m, which is the current legal limit in most countries without the need for some special exemption [1].

The experiments were carried out using the Keras library (keras.io, version 2.2.4) with TensorFlow v. 1.4. Keras library had available all models used in the experiments, thus avoiding the need for direct coding or using third-party sources. With this setup, most CNNs could be trained using images of any dimension as input, even when using pretrained networks (transfer learning), as was the case in this study. However, there are some architectures that require the input dimensions to be either a fixed or above a certain lower limit (see Table 1). In cases like these, it is necessary to upsample the images after downsampling to meet the requirements of those architectures. It is important to emphasize that a direct comparison between architectures is still possible even if the input dimensions are different because the downsampling causes a loss of information that is not reverted by the upsampling.

Fifteen different CNN architectures were tested (Table 1) using the following hyperparameters: fixed learning rate of 0.0001, 10 epochs (most models converged with fewer than five epochs), mini-batch size of 128 (larger values caused memory problems with deeper architectures) and sigmoid activation function. Transfer learning was applied by using models pretrained on the Imagenet dataset [25] and freezing all convolutional layers and updating only the top layers. Training was performed in a workstation equipped with two RTX-2080 Ti GPUs.

Finally, model assessment was carried out by taking the trained models and applying them to the independent test sets. Four performance metrics were extracted:

(1) Accuracy=(TP+TN)/(TP+TN+FP+FN),

(2) Precision=TP/(TP+FP),

(3) Recall=TP/(TP+FN),

(4) F1Score=2×(Recall×Precision)/(Recall+Precision),

where TP, TN, FP and FN are the number of true positives, true negatives, false positives and false negatives, respectively.

## 3. Results

Table 2 summarizes the results obtained having the first dataset as reference (“cattle” image blocks centered exactly at the position of each animal). The objective of this experiment was to assess the performance of the models when trained with samples carefully generated to represent each class as consistently as possible. Only the 224 × 224 pixel samples were used in this case because this experiment was carried out only to serve as a reference for the more realistic one shown in Table 3.

**Figure 7:**
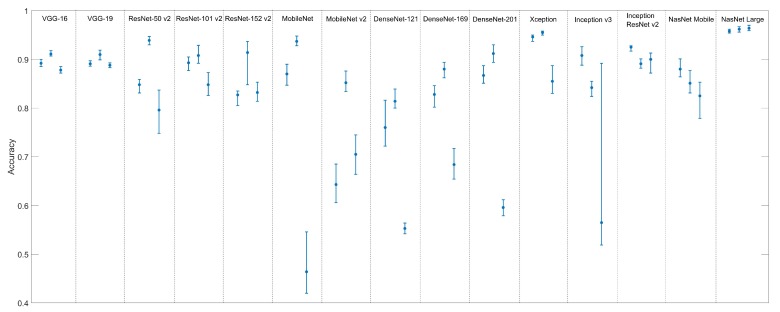
Range of accuracies obtained for each CNN architecture. The three bars associated to each architecture correspond to input sizes of 224 × 224 (**left**), 112 × 112 (**middle**) and 56 × 56 (**right**). The circle in each bar represents the average accuracy, and the bottom and top extremities represent the lowest and highest accuracies observed during the application of the 10-fold cross-validation.

Table 3 is similar to Table 2, except the samples used in the training were generated using the second dataset (regular grid). This is a more challenging situation, as some “cattle” image blocks may contain only small parts of the animals which need to be properly detected by the models. In addition, Figure 7 shows graphically the average and range of accuracies obtained for each model.

Table 4 shows the average time that it took to train each CNN over one epoch.

## 4. Discussion

The results shown in Table 2 reveal that most models were able to reach accuracies above 95% when training and test image blocks were carefully generated to provide the best characterization of each class. Very deep structures, like NasNet Large, were able to yield accuracies close to 100%. In practice, using models trained this way is challenging: the positions of the animals in a new image to be analyzed by the model will not be known a priori (otherwise the problem would be solved already), so image blocks cannot be properly generated to perfectly encompass the animals to be detected. One possible solution to this problem is to sweep the entire image using a sliding window in such a way each animal will appear, at least partially, in multiple image blocks to be analyzed by the model. This approach, which has been explored by [15,17,18], enables the construction of a heat map showing the likely positions of the animals in the image. The problem with this approach is that the number of image blocks to be analyzed may be very high; on the other hand, applying the models is much faster than training them, which makes this approach adequate in many situations.

Overall, CNNs were remarkably robust to almost all variations in illumination conditions. No noticeable differences in accuracy were observed when only images with illumination issues (insufficient and excessive brightness, shadows) were considered. The only exception was with the presence of severe specular reflection, in which case the contrast between animals and ground tended to become too slight for the models to detect then correctly. Excessively blurred images also caused problems. Specular reflection and blur explain most of the errors observed when the dataset labeled with exact animal locations was used. Models like NasNet Large and the ResNet were fairly robust even under these poor conditions. Those models did not reach 100% accuracy because, in a few instances, the number of problematic images in the test set was proportionally much higher, a consequence of the randomness of the division process.

**Figure 8:**
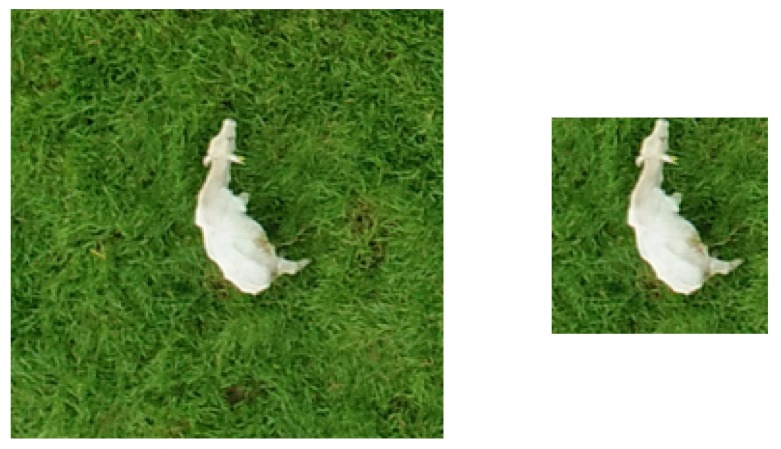
Examples of the two possible approaches to simulate coarser ground sample distances (GSDs). In the left, the entire image is downsampled and then the regular grid is applied; in the right, the grid is first applied and then image blocks are downsampled.

As expected, accuracies were lower when the regular grid was used. Interestingly, for 12 of the 15 models, the best results were achieved using the 112 × 112 input size. This indicates that the ideal GSD for cattle detection is 2 cm/pixel. At a first glance, this may seem counterintuitive, because higher resolutions tend to offer more information to be explored by the models. However, the way most CNNs architectures are designed (filter and convolution sizes), features related to cattle can be more effectively extracted at this GSD. As an added bonus, these results indicate that we could fly twice as high (thus covering a much larger area) without losing any accuracy. It is worth mentioning that another method for simulating different GSDs was considered, in which the entire image is downsampled and then the regular grid is applied, instead of first applying the grid and then downsampling the image blocks. The main difference between both approaches is that in the latter the relative area occupied by animals within each block is kept the same, while in the former the area occupied by the animals will decrease in the same proportion as the downsampling. In both cases, the number of pixels associated with the animals will be exactly the same, but in the approach adopted here the amount of background will be much lower (Figure 8). Some limited experiments were performed using the alternative approach, and a drop in accuracy was observed for all models but Inception v3, for which the accuracy was slightly better.

**Figure 9:**
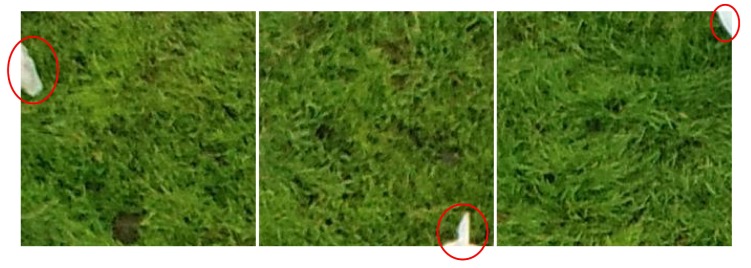
Examples of “cattle” blocks with very small animal parts visible (red elipses).

The drop in accuracy observed when the regular grid approach was adopted is almost exclusively due to many image blocks annotated as “cattle” having very few pixels actually associated with an animal (Figure 8). Because such blocks contain mostly background, it may be difficult for the model to learn the correct features associated with cattle during training—if the wrong features are learned, the potential for misclassifications rises considerably. The reverse problem occurs during the application of the model: since the background is so dominant, the number of pixels associated with animals may not be enough for proper detection. However, considering the amount of “cattle” blocks showing only a small fraction of the animals’ bodies (Figure 9), it is remarkable that some models were able to yield accuracies above 95%. It is also important to remark that errors of this kind are not very damaging: blocks with small animal parts will almost certainly have one or more neighboring blocks containing the remainder of the animal, in which case correct detection is more likely. Errors caused by missed small parts can be compensated by other image processing techniques, which can be used to refine the delineation of the animals. There are some approaches that tackle this problem explicitly, like region proposal networks [4] and the concept of anchor boxes used in YOLO [7]. These alternative techniques will be investigated in future experiments.

Most CNN architectures performed well under the experimental conditions of this study. Taking only accuracy into consideration, the most successful CNN was NasNet Large. The very deep and complex structure associated with this architecture made it very robust to all the challenging conditions found in the dataset used in the experiments. The Xception architecture had slightly lower accuracies, especially when the 112 × 112 input size was used, but its training time was several times faster than that associated with NasNet large. Among the lightest architectures (mostly developed for use in mobile devices), MobileNet (version 1) showed the best performance. All trained models took less than one second to fully analyze a 4864 × 3648-pixel image when ran in the workstation mentioned in Section 2.2. Operational time differences between models are expected to increase when devices with less computational power are used (e.g., mobile devices), but testing the models under more restricted setups was beyond the scope of this study.

As mentioned before, most of the experiments were performed with animals of the Canchim breed, whose colors range from white to light beige, with some darker coating occurring in some animals. Because of its Nelore roots, it shares many visual characteristics with this breed, and as a result, the models generated in this work were also effective in detecting Nelore animals. Other breeds would probably require the training of new models, and the degree of success that can be potentially achieved in each case would have to be investigated. However, taking into consideration the results reported in the literature for other breeds and other experimental setups, it seems evident that deep learning architectures are remarkably successful in extracting relevant information that can lead to accurate detection of cattle in images captured by UAVs.

## 5. Conclusions

This article presented a study on the use of deep learning models for the detection of cattle (Canchim breed) in UAV images. The experiments were designed to test the robustness of 15 different CNN architectures to factors like low illumination, excessive brightness, presence of blur, only small parts of the animal body visible, among others. Most models showed a remarkable robustness to such factors, and if a few precautions are taken during the imaging missions, accuracy rates can be close to 100%. In terms of accuracy, NasNet Large yielded the best results, with the Xception architecture also producing very high accuracies with a faction of NasNet’s training times. The most accurate results were obtained with a GSD of 2 cm/pixel, indicating that images can be captured at relatively high flight altitudes without degrading the results. Future work will focus on testing new approaches for animal detection. In particular, future experiments will investigate how techniques that generate a bounding box around the objects of interest (e.g. YOLO) and semantic segmentation methods perform under the same conditions tested in this work. At the same time, the image dataset will continue to receive new images, thus expanding, even more, the variety of conditions. Breeds other than Canchim are also expected to be included. The image database used in this experiment is currently unavailable for external researchers, but this will likely change as soon as the database is properly organized and some restrictions are lifted.

## Figures and Tables

**Table 1:** Convolutional neural networks (CNNs) architectures tested in the experiments.

CNN Architecture	Required Input Size	Reference
VGG-16	None	Simonyan and Zisserman [26]
VGG-19	None	Simonyan and Zisserman [26]
ResNet-50 v2	None	He et al. [27]
ResNet-101 v2	None	He et al. [27]
ResNet-152 v2	None	He et al. [27]
MobileNet	None	Howard et al. [28]
MobileNet v2	None	Sandler et al. [29]
DenseNet 121	None	Huang et al. [30]
DenseNet 169	None	Huang et al. [30]
DenseNet 201	None	Huang et al. [30]
Xception	≥75 × 75 pixels	Chollet et al. [31]
Inception v3	≥75 × 75 pixels	Szegedy et al. [32]
Inception ResNet v2	≥75 × 75 pixels	Szegedy et al. [33]
NASNet Mobile	224 × 224 pixels	Zoph et al. [34]
NASNet Large	331 × 331 pixels	Zoph et al. [34]

**Table 2:** Results obtained using the dataset labelled with exact animal locations.

CNN	Accuracy	Precision	Recall	F1 Score
VGG-16	0.972	0.973	0.973	0.970
VGG-19	0.973	0.973	0.973	0.975
ResNet-50 v2	0.977	0.978	0.978	0.975
ResNet-101 v2	0.983	0.985	0.985	0.985
ResNet-152 v2	0.967	0.970	0.970	0.965
MobileNet	0.983	0.980	0.980	0.983
MobileNet v2	0.787	0.855	0.790	0.778
DenseNet 121	0.852	0.895	0.868	0.865
DenseNet 169	0.935	0.943	0.933	0.935
DenseNet 201	0.935	0.945	0.938	0.938
Xception	0.969	0.968	0.968	0.968
Inception v3	0.979	0.975	0.975	0.975
Inception ResNet v2	0.983	0.983	0.983	0.985
NASNet Mobile	0.857	0.890	0.858	0.853
NASNet Large	0.992	0.993	0.993	0.995

**Table 3:** Results obtained using the dataset labelled with exact animal locations.

CNN	Input Size	Accuracy	Precision	Recall	F1 Score
	224 × 224	0.892	0.903	0.895	0.893
VGG-16	112 × 112	0.911	0.918	0.915	0.910
	56 × 56	0.878	0.888	0.880	0.878
	224 × 224	0.891	0.900	0.893	0.890
VGG-19	112 × 112	0.910	0.915	0.910	0.908
	56 × 56	0.888	0.898	0.885	0.885
	224 × 224	0.848	0.883	0.848	0.845
ResNet-50 v2	112 × 112	0.939	0.940	0.943	0.940
	56×56	0.796	0.815	0.793	0.788
	224 × 224	0.893	0.910	0.895	0.890
ResNet-101 v2	112 × 112	0.908	0.913	0.908	0.910
	56 × 56	0.848	0.875	0.850	0.845
	224 × 224	0.827	0.868	0.820	0.815
ResNet-152 v2	112 × 112	0.914	0.890	0.885	0.890
	56 × 56	0.832	0.865	0.835	0.830
	224 × 224	0.870	0.898	0.870	0.868
MobileNet	112 × 112	0.937	0.943	0.938	0.938
	56 × 56	0.464	0.508	0.485	0.450
	224 × 224	0.643	0.793	0.648	0.595
MobileNet v2	112 × 112	0.852	0.888	0.855	0.853
	56 × 56	0.705	0.755	0.705	0.690
	224 × 224	0.760	0.840	0.773	0.755
DenseNet 121	112 × 112	0.814	0.865	0.820	0.813
	56 × 56	0.553	0.763	0.555	0.448
	224 × 224	0.828	0.873	0.828	0.823
DenseNet 169	112 × 112	0.880	0.900	0.878	0.875
	56 × 56	0.684	0.778	0.688	0.658
	224 × 224	0.867	0.898	0.873	0.868
DenseNet 201	112 × 112	0.912	0.923	0.913	0.910
	56 × 56	0.596	0.773	0.598	0.518
	224 × 224	0.946	0.948	0.943	0.943
Xception	112 × 112	0.955	0.953	0.953	0.955
	56 × 56	0.855	0.865	0.858	0.855
	224 × 224	0.908	0.908	0.910	0.908
Inception v3	112 × 112	0.842	0.843	0.838	0.838
	56 × 56	0.565	0.740	0.703	0.648
	224 × 224	0.925	0.933	0.925	0.923
Inception ResNet v2	112 × 112	0.891	0.890	0.890	0.890
	56 × 56	0.900	0.898	0.895	0.890
	224 × 224	0.880	0.905	0.883	0.880
NASNet Mobile	112 × 112	0.851	0.885	0.855	0.850
	56 × 56	0.825	0.858	0.818	0.810
	224 × 224	0.958	0.963	0.960	0.958
NASNet Large	112 × 112	0.962	0.963	0.963	0.963
	56 × 56	0.964	0.965	0.965	0.965

**Table 4:** Average training time for different input sizes (in seconds per epoch).

CNN Architecture	224 × 224	112 × 112	56 × 56
VGG-16	65	14	6
VGG-19	74	17	6
ResNet-50 v2	41	11	7
ResNet-101 v2	68	19	8
ResNet-152 v2	94	27	12
MobileNet	18	8	7
MobileNet v2	20	8	7
DenseNet 121	56	15	9
DenseNet 169	65	18	10
DenseNet 201	81	22	11
Xception	60	13	-
Inception v3	29	9	-
Inception ResNet v2	72	19	-
NASNet Mobile	35	-	-
NASNet Large	334 *	-	-

* NasNet only runs with 331x331 input size.
